# A European field assessment of the efficacy of fluralaner (Bravecto^®^) chewable and spot-on formulations for treatment of dogs with generalized demodicosis

**DOI:** 10.1186/s13071-020-04159-2

**Published:** 2020-06-11

**Authors:** Ivo Petersen, Rafael Chiummo, Eva Zschiesche, Joanna Karas-Tecza, Dhimiter Rapti, Rainer Roepke, Emmanuel Thomas

**Affiliations:** 1 MSD Animal Health Innovation GmbH, Zur Propstei, 55270 Schwabenheim, Germany; 2 Dermatology Clinic for dogs and cats, Dermawet, Warsaw, Poland; 3 Faculty of Veterinary Medicine, Agriculture University of Tirana, Tirana, Albania

**Keywords:** Bravecto^®^, *Demodex*, Dog, Field study, Fluralaner, Generalized demodicosis

## Abstract

**Background:**

Recent reports indicate that the isoxazoline compounds have the potential to provide safe and effective treatment of canine generalized demodicosis, a condition that has been traditionally difficult to cure. Controlled field studies are needed to confirm this potential. A study was therefore initiated to investigate the efficacy of a single oral or spot-on treatment with fluralaner, an isoxazoline, compared with multiple topical treatments with imidacloprid-moxidectin, in dogs naturally affected by generalized demodicosis.

**Methods:**

Veterinary clinics in 5 European countries enrolled 134 dogs diagnosed with generalized demodicosis. Dogs were randomized to treatment with either fluralaner chewables, fluralaner spot-on, or topical imidacloprid-moxidectin in a 2:2:1 ratio. Both fluralaner formulations were administered once, at the approved dose rate, on Day 0. Imidacloprid-moxidectin was administered per label on Day 0, and every 4 weeks, more frequently if necessary. At each visit (Days 0, 28, 56, 84), dogs were monitored for demodectic mites using deep skin scrapings and observed for health and for severity of skin lesions. Treatment was considered efficacious if more than 90% of the dogs were free of live mites at both Days 56 and 84.

**Results:**

Of 124 dogs completing the study, 57 were diagnosed with juvenile-onset demodicosis and 67 with the adult-onset form. A single treatment with oral or spot-on fluralaner was efficacious, each eliminating mites from at least 98.0% of treated dogs on Days 56 and 84. Against juvenile-onset demodicosis, efficacy of the oral and spot-on formulations was 96.0% and 100%, respectively, and against adult-onset demodicosis 100% and 96.7%. Multiple administrations of imidacloprid-moxidectin were not efficacious, eliminating mites from 87.5% of dogs (92.0% with juvenile-onset demodicosis cured; 81.8% with adult-onset demodicosis). All groups showed a marked reduction in skin lesions by Day 28, with continuing clinical improvement at each subsequent visit through Day 84. There were no treatment-related adverse events.

**Conclusions:**

A single administration of fluralaner chewables or fluralaner spot-on is highly effective against with juvenile-onset and adult-onset forms of generalized canine demodicosis. Topically applied imidacloprid-moxidectin at weekly to monthly intervals over the 84-day study did not achieve the proportion of mite-free dogs required to demonstrate efficacy. 
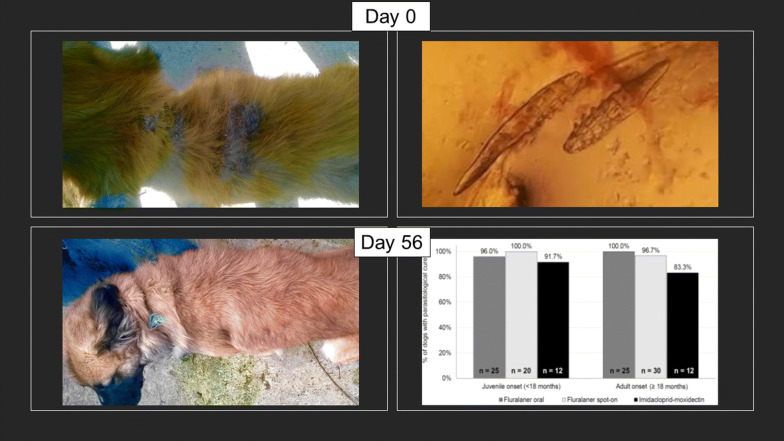

## Background

The mite *Demodex canis*, regarded as a normal inhabitant of the canine integument, is passed from the bitch to her nursing puppies by direct contact [1, 2]. The mites spend their entire life-cycle in the lumen of the hair follicles, although in heavy infestations, may also invade sebaceous glands [3]. Female mites lay eggs that develop into eight-legged, slender cigar-shaped adults within approximately 3–4 weeks [3]. In some dogs there is an abnormal proliferation of mites which can manifest as a localized alopecia that resolves either spontaneously or following acaricidal treatment. In a smaller proportion of dogs, possibly with a hereditary predisposition, underlying disease (e.g. hyperadrenocorticism, hypothyroidism, diabetes mellitus, neoplasia) or treatment-related immunosuppression (e.g. corticosteroids, chemotherapeutic agents), the infestation persists, and potentially becomes generalized and life-threatening [2]. Generalized demodicosis can occur in dogs aged from 2 to 18 months (juvenile demodicosis), or as an adult-onset form in mature dogs [2]. Dogs with generalized demodicosis may develop severe pustular lesions because of secondary infections, leading to deep pyoderma, furunculosis and cellulitis. At this point the treatment, often lasting 6 to 8 weeks, can only be successful if the mites are eradicated [2, 3].

Until recently, products approved for the treatment of generalized demodicosis have required topical or oral administration daily, at weekly or 2-weekly intervals, or at monthly intervals for up to six or more months, with the frequency and duration of treatments governed by the clinical response and progress to parasitological cure [4–9]. Recent reports indicate that orally administered isoxazoline compounds can provide safe and effective treatment of generalized canine demodicosis, even with very severe presenting signs, and three of these compounds, sarolaner, afoxolaner and fluralaner, are now approved in Europe for this indication [10–17].

The extended activity of fluralaner with an efficacy duration of up to 12 weeks, against fleas and ticks, offers potential for successful cure of generalized demodicosis following a single treatment [18, 19]. Two laboratory studies showed that a single administration of either an oral or spot-on formulation of fluralaner to dogs affected by generalized demodicosis resulted in elimination of *Demodex* spp. mites, based on deep skin scrapings, and resolution of clinical signs. A non-blinded, uncontrolled, open-label study with orally administered fluralaner provided evidence that those laboratory results could translate into field efficacy [10, 16, 17].

An investigator-masked, positive controlled, multicenter study was initiated to provide field-based confirmation of the efficacy of single treatment with fluralaner chewable tablets or fluralaner spot-on against canine generalized demodicosis. The study was conducted in Europe to support a label indication for fluralaner for the treatment of dogs presenting with the disease.

## Methods

This study was conducted following Good Clinical Practices, VICH Guideline 9 [20], Guideline on Statistical Principles for Veterinary Clinical Trials [21], and Guideline for the Demonstration of Efficacy of Ectoparasiticides [22]. Seven veterinary practices across Albania, Poland, Spain, Germany and Portugal enrolled client-owned dogs presented with generalized demodicosis.

### Enrollment of dogs

Generalized demodicosis was diagnosed by the presence of more than 4 affected skin areas with lesion diameter > 2.5 cm, or pododemodicosis in at least one paw; with positive skin scrapings (≥ 3 *Demodex* spp. mites) [2, 11, 14]. Dogs were at least 8 weeks-old and weighed at least 2.0 kg. Any dog needing intensive care, or that had been treated with injectable corticosteroids in the past 30 days was excluded. Ongoing treatment with oral or topical corticosteroids had to be discontinued before inclusion in the study. Dogs were excluded if treated with: fluralaner in the previous 3 months; other isoxazoline products within 35 days; macrocyclic lactone parasiticides, except at approved heartworm prevention doses, within 30 days; amitraz, fipronil, pyriproxyfen, metaflumizone, pyrethrins, deltamethrin or permethrin, within 30 days; or shorter-acting products with miticidal activity within 14 days. Treatment of enrolled dogs with corticosteroids, immunosuppressants (cyclosporine, oclacitinib) or with any product having miticidal activity was not permitted during the study, and owners were instructed to avoid using in-house or on-property acaricides, and to avoid shampooing the study dog during the 3 days following each treatment. Each owner was also instructed to observe their dog for any unfavorable or unexpected events, and to report any such observations to the study clinic. Enrolled dogs were maintained in their home environment throughout the study, and owners returned their dog to the clinic on Days 28, 56 and 84.

### Treatments

Qualifying dogs enrolled in each clinic were randomly allocated to either a fluralaner group (oral or spot-on) or to a topical imidacloprid-moxidectin group in a ratio of 2:2:1.

Dogs in the oral fluralaner group received a single fluralaner chewable (Bravecto^®^ chewable tablets, MSD Animal Health, Schwabenheim, Germany) administered at the label dose rate of 25–56 mg/kg. These dogs were fed within one hour of treatment and observed for 10 min after treatment to verify that the tablets were retained. In the other fluralaner group, dogs received a single spot-on 28% w/v fluralaner application (Bravecto^®^ Spot-On Solution, MSD Animal Health), at the label dose rate of 25–56 mg/kg. Dogs in the imidacloprid-moxidectin group received multiple sequential applications of at least 10 mg/kg imidacloprid and 2.5 mg/kg moxidectin (Advocate^®^ for Dogs, Bayer). In this group, dogs with mild to moderate demodicosis were treated every 4 weeks, while at the discretion of the dispensing veterinarian the treatment frequency of severely affected dogs could be increased to up to once per week. All products were administered according to the product prescribing information directions. Fluralaner treatment and the initial imidacloprid-moxidectin treatment were administered on Day 0. When clinical signs of *Demodex*-associated pyoderma were observed, study dogs could receive antibiotics or topical antiseptics at the discretion of the attending veterinarian.

### Assessments of demodicosis

At each scheduled visit, deep skin scrapings for *Demodex* mites were made at five different skin areas, each scraping approximately 1 cm^2^. Where necessary (e.g. long or medium-haired dogs), hair was removed at the area to be scraped, and the skin was firmly squeezed prior to and during scraping to eject mites from hair follicles [2, 14, 15]. Scrapings were made in the direction of hair growth with a blade or spatula, covered with mineral oil, until capillary oozing was observed. Hairs were plucked from affected areas where obtaining a scraping was difficult (e.g. periocular and interdigital areas). Efforts were made to scrape the same affected areas at all assessments, unless those areas appeared normal and other active lesions were present. The collected material was transferred to a slide, mixed with mineral oil, placed under a cover slip and examined under a microscope (4× or 10× objective) to count live mites (larvae, nymphs and adult stages) (eggs were not counted). Clinical signs and extent of demodectic lesions on each dog were assessed on the days on which scrapings were made. Dermatological signs of alopecia, erythema, crusts, scales and papules were assessed and graded absent, mild, moderate or severe. Estimates were made of the overall extent of skin lesions using the following scale: 0, no lesions; 1–9% of the body affected; 10–29% of the body affected; 30–49% of the body affected; and ≥ 50% of the body affected [14].

### Statistical analysis

Primary efficacy was determined for each study group using the percentage of dogs free of live mites at the both of the last two evaluation time points (Days 56 and 84). Treatment was considered efficacious if the percentage of animals that were free of live mites at both evaluation time points exceeded 90%. The percentage of dogs free of live mites was also calculated separately for each visit. A descriptive analysis was performed for mite counts and skin lesions: the distribution of mite counts and the number of dogs free of skin lesions were determined at each visit, as well as the distribution of types and extent of skin lesions.

## Results

Across Albania (*n* = 60), Germany (*n* = 3), Spain (*n* = 7), Portugal (*n* = 14), and Poland (*n* = 50), 134 dogs were enrolled, ranging in age in the oral fluralaner group from 10 weeks to 13 years (mean 3.1; standard deviation ± 2.9 years), in the spot-on fluralaner group from 4 months to 12 years (3.5 ± 3.2 years), and in the topical imidacloprid-moxidectin group from 11 weeks to 9 years (2.9 ± 2.9 years). A broad range of breed categories was represented, with approximately 50% of dogs mixed breed, 22% were companion-toy dogs, and approximately 9% were terriers.

During the study, of the 134 dogs that were enrolled, 10 became ineligible for inclusion in study efficacy assessments. Four dogs in the fluralaner spot-on group, two in the fluralaner chewable group and one dog in the imidacloprid-moxidectin group were excluded because of an owner failure to adhere to follow-up visit schedules. Two dogs were excluded because of treatment with study-prohibited drugs: one dog in the fluralaner chewable group received oclacitinib and dexamethasone, and a dog in the fluralaner spot-on group received dexamethasone. The death prior to Day 56 of a fluralaner spot-on group dog that had shown weakness, anorexia, dyspnea and dehydration, was diagnosed as cardiac failure, unrelated to treatment. Thus, data from 124 dogs were available for determination of product efficacy, 50 in each of the fluralaner groups and 24 in the imidacloprid-moxidectin group. In the latter group most dogs received 4 treatments (once every 4 weeks), 5 dogs required 3 treatments; 2 dogs received 13 treatments (one per week); one received 10; and one received 7 treatments. In the fluralaner chewable group 25 dogs showed juvenile-onset demodicosis and 25 dogs showed adult-onset demodicosis. In the fluralaner spot-on group 20 dogs showed juvenile-onset demodicosis and 30 dogs adult-onset demodicosis, and in the imidacloprid-moxidectin group 12 dogs showed juvenile-onset demodicosis and 12 dogs adult-onset demodicosis.

On Days 56 and 84, in both fluralaner groups 98.0% of dogs had shown a parasitological cure (free of mites at two consecutive assessments) (Table 1). In the imidacloprid-moxidectin group, the percentage of mite-free dogs at both Days 56 and 84 was 87.5%, below the pre-determined efficacy threshold of 90%. On Day 84, 100% of fluralaner chewable and 98% of fluralaner spot-on treated dogs were free of live mites, as were 91.7% of dogs in the topical imidacloprid-moxidectin group.

**Table 1:** Number (%) of dogs treated once on Day 0 with fluralaner chewables or fluralaner spot-on or on multiple occasions with topical imidacloprid-moxidectin that were negative for live mites on Days 28, 56 and 84

	Fluralaner		Imidacloprid-moxidectin
	Oral (*n* = 50)	Spot-on (*n* = 50)	Topical (*n* = 24)
Mite free at Day 28	46 (92.0)	46 (92.0)	19 (79.2)
Mite free at Day 56	49 (98.0)	49 (98.0)	21 (87.5)
Mite free at Day 84	50 (100)	49 (98.0)	22 (91.7)
Mite free at Days 56 and 84	49 (98.0^a^)	49 (98.0)	21 (87.5)

^a^One dog treated with fluralaner chewable had 500 mites on Day 0, 52 on Day 28, 1 mite on Day 56 and 0 on Day 84 and had improved condition. This dog was considered a treatment failure because it was not free of mites on both Day 56 and Day 84

Mean mite counts on Day 0 in the fluralaner spot-on and chewable groups were 53.2 and 30.4, respectively, and 37.8 in the topical imidacloprid-moxidectin group (Table 2). For the 25 dogs in the fluralaner oral group that presented with juvenile-onset demodicosis, all but one were free of mites on both Days 56 and 84 (efficacy 96.0%) (Table 3). For the fluralaner spot-on and imidacloprid-moxidectin groups, the equivalent efficacy against juvenile-onset demodicosis was 100% (20 of 20 dogs free of mites on Days 56 and 84) and 91.7% (11 of 12 dogs), respectively. For efficacy against adult-onset demodicosis, in the fluralaner oral group 25 of 25 dogs (100%) were free of mites on both Days 56 and 84, as were 28 of 29 (96.7%) dogs treated with spot-on fluralaner, and 10 of 12 dogs (83.3%) in the imidacloprid-moxidectin group.

**Table 2:** Mite counts (mean ± standard deviation) in dogs with generalized demodicosis, treated once on Day 0 with fluralaner chewables or fluralaner spot-on, or on multiple occasions with topical imidacloprid-moxidectin

Day	Fluralaner		Imidacloprid-moxidectin
	Oral (*n* = 50)	Spot-on (*n* = 50)	Topical (*n* = 50)
0	53.2 ± 80.3	30.4 ± 21.9	37.8 ± 28.8
28	1.1 ± 7.4	0.4 ± 2.2	1.2 ± 4.1
56	0.0 ± 0.1	0.0 ± 0.1	0.3 ± 0.7
84	0.0 ± 0.0	0.0 ± 0.1	0.1 ± 0.3

**Table 3:** Percentage of dogs (number cured/number affected on Day 0) with a parasitological cure of juvenile- or adult-onset demodicosis (free of mites on Days 56 and 84) following a single treatment with oral or topical fluralaner or multiple treatments with topical imidacloprid-moxidectin

	Oral fluralaner	Topical fluralaner	Topical imidacloprid-moxidectin
Juvenile-onset demodicosis	96.0 (24/25)	100 (20/20)	91.7 (11/12)
Adult-onset demodicosis	100 (25/25)	96.7 (29/30)	83.3 (10/12)

**Fig. 1:**
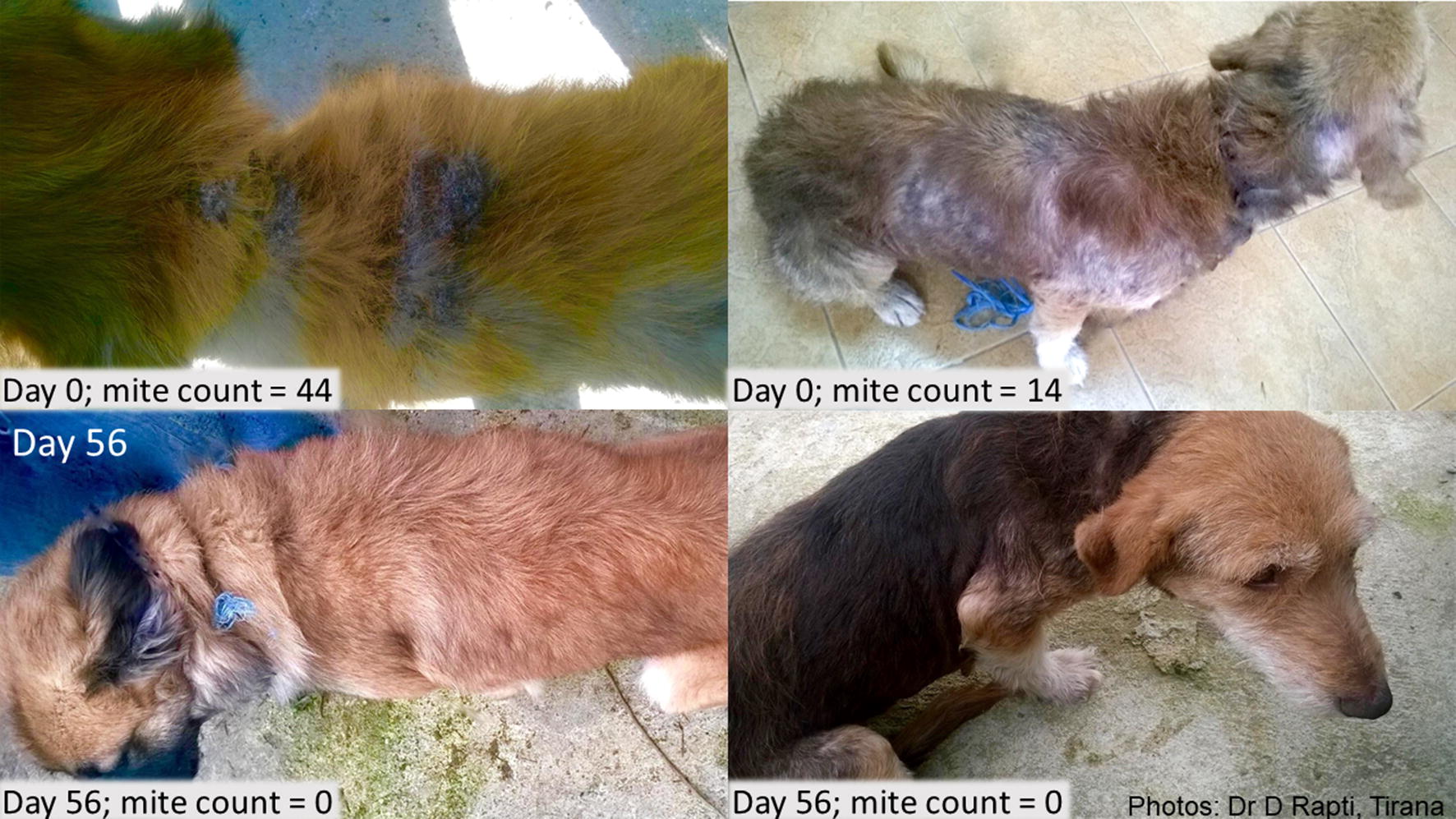
Photograph on Day 0 of dogs with demodicosis-related dermatological lesions, and Day 56 showing hair regrowth following a single fluralaner treatment on Day 0 (left, fluralaner spot-on; right, fluralaner chewable)

At the first visit (Day 0), all enrolled dogs had clinical signs of generalized demodicosis, most commonly alopecia which was present in all study dogs. By Day 84, there was no observable alopecia in 94.0% of oral fluralaner-treated dogs, 84.0% of spot-on fluralaner-treated dogs, and 75.0% of topical imidacloprid-moxidectin treated dogs. Other initial clinical signs included crusts (61.3% of dogs), erythema (58.9%), scales (34.7%), papules (26.6%), pustules (13.7%), ulcerations (9.7%) and comedones (4.0%). All signs of erythema, comedones, papules, pustules and ulcerations resolved in dogs in all groups by Day 84. Overall, there was a marked reduction in skin lesions in all groups at Day 28, with substantial hair regrowth by Day 56 (Fig. 1). Continuing improvement was observed at each subsequent visit through the final visit on Day 84.

No treatment-related adverse events were observed in any dogs in any of the three study groups.

## Discussion

These results show that under field conditions a single oral or spot-on administration of fluralaner is effective in the treatment of generalized demodicosis. Evidence of the potent fluralaner effect, in both oral and spot-on formulations, was present as early as 28 days following treatment, when 92.0% of dogs were found to be free of live mites. In comparison, topical imidacloprid-moxidectin did not reach the pre-defined efficacy threshold for generalized demodicosis. At the Day 28 assessment, 79.2% of imidacloprid-moxidectin treated dogs were live-mite free, indicating that the miticidal onset was not reaching the 90% threshold at this time. Both fluralaner formulations were effective in the treatment of generalized juvenile- and adult-onset manifestations of demodicosis. Multiple topical applications of imidacloprid-moxidectin did not achieve the required level of mite elimination to be effective as a treatment of the adult-onset disease.

To our knowledge, this is the first controlled field study with fluralaner, and just the second controlled field study with any isoxazoline, to demonstrate field efficacy in the treatment of generalized demodicosis. In the only other controlled isoxazoline field study, in Europe, dogs with generalized demodicosis were treated orally at monthly intervals with sarolaner at a dose rate of 2–4 mg/kg, or with imidacloprid-moxidectin as used in the present study [14]. At Day 30, 15.1 and 17.9% of dogs treated with sarolaner or imidacloprid-moxidectin, respectively, were mite-free, respectively, while in the present study, at the equivalent time point (Day 28), 92.0% of dogs treated with a fluralaner formulation were mite-free, and 79.2% of dogs in the imidacloprid-moxidectin group were free of mites. The sarolaner study concluded 180 days after the first treatment, with up to six administrations that drug producing a parasitological cure in all dogs that remained in the study, and up to 24 weekly doses of imidacloprid-moxidectin producing a parasitological cure in 91.7% of the remaining dogs in that group [14]. In the present study, a parasitological cure was achieved by Day 84 in 98.0% of dogs treated once with a fluralaner formulation, but in only 87.5% of dogs treated with imidacloprid-moxidectin. Overall, the results of these studies indicate that the isoxazoline compounds, particularly the single-treatment fluralaner formulations, offer an advance over earlier treatments for canine generalized demodicosis.

In an uncontrolled study in Europe, two formulations of afoxolaner for oral administration, one as a single entity product, the other in combination with milbemycin oxime, were administered on Days 0, 28 and 56 to dogs presenting with generalized demodicosis [15]. On Day 28, 6 of 50 (12.0%) treated dogs were mite-free; on Day 56, of 49 dogs remaining in the study, 19 (38.8%) were mite-free. Thus, the maximum possible Day 84 cure rate (two consecutive assessments in which dogs were mite-free) following the three afoxolaner treatments was 38.8%. In the sarolaner study described above, assuming the dogs that were mite-free on Day 60 maintained that status through Day 90, the parasitological cure rate would have been 69.2% [14]. That cure rate compares favorably with the Day 84 cure rate reported from the afoxolaner study [15], but not with the cure rate in the fluralaner study reported herein (98.0%). Taken together, the results of the three studies suggest that the onset of parasitological effect of fluralaner against *D. canis* may be observed more quickly than with either of the other field-tested isoxazolines, and the long-term effect more complete following a single fluralaner treatment than following up to six treatments with a shorter acting isoxazoline.

In another uncontrolled field study involving dogs with generalized demodicosis, in Thailand, a parasitological cure was observed in 100% of dogs (*n* = 67) within three months following treatment with orally administered fluralaner [16]. This is consistent with our finding of a Day 84 parasitological cure in 100% of dogs treated orally with fluralaner and 96.1% of those receiving fluralaner spot-on. An extended duration of follow up in that study found that 4 of 46 successfully treated adult-onset dogs had relapsed at 2, 7, 10 or 12 months following cure. The possibility of relapse, as long as 12 months after successful treatment, emphasizes the need for extended post-cure vigilance, particularly of older dogs, in which a cure has been realized.

Six laboratory studies, all from a single facility in South Africa, have shown the potential of each of the isoxazolines in the treatment of canine generalized demodicosis [10–13, 17, 23]. Three consecutive treatments at 4-weekly or monthly intervals were used in separate studies of sarolaner [11] and afoxolaner [12], and in an uncontrolled study with lotilaner [13]. Two studies, one of which was uncontrolled, investigated single-treatment fluralaner efficacy using the chewable formulation [10, 23], and one controlled study involved the spot-on formulation [17]. The multiple treatments with afoxolaner, sarolaner and lotilaner, and the single treatment controlled study with either fluralaner formulation, all reduced mean demodectic mite counts by > 99% within the month following the Day 0 treatment, and by 100% at Days 81 or 84. Across these studies, the reported efficacies of imidacloprid-moxidectin within the month after the first treatment ranged from 9.8 to 89.8%, and on Day 84, following multiple treatments, from 0 to 100%. In the uncontrolled oral fluralaner study, PCR analysis of hair collected from treated dogs found that *Demodex* DNA levels progressively declined throughout the study, and on Day 112 were reduced 1000-fold from pre-treatment levels [23]. Thus, the accumulating data in which the isoxazolines have shown potent efficacy and superiority over imidacloprid-moxidectin indicate that they would be the treatment of choice for generalized demodicosis, although further controlled studies would be helpful to confirm that the miticidal efficacy of afoxolaner and lotilaner is maintained under field conditions.

Regardless of the efficacy of these products, generalized demodicosis, particularly in older dogs, becomes clinically apparent in those affected by another disease process, or by immunosuppressive therapy, factors which can also predispose successfully treated dogs to a relapse [2, 5, 16]. A corollary of that risk of relapse is the requirement for continued vigilance of cured dogs, and the associated potential need for repeated miticidal treatments over an extended period. In this regard, the long efficacy duration of fluralaner has been linked to better owner adherence compared with use of monthly treatments [24]. The sustained action and convenience of fluralaner formulations could also help to improve the long-term cure rate of dogs with generalized demodicosis.

## Conclusions

Fluralaner (Bravecto^®^) chewable and spot-on formulations were highly efficacious in providing a parasitological and clinical cure in dogs affected with generalized canine demodicosis over a period of 12 weeks following a single administration at a dose rate of 25–56 mg/kg.

## Data Availability

The datasets generated and analysed during the present study are not publicly available due to confidentially agreements. All original study documentation is archived by the sponsor at MSD facilities (Germany).

## Abbreviations

VICH: Veterinary International Cooperation on Harmonisation
